# Revealing and Tuning the Photophysics of C=N Containing Photothermal Molecules: Excited State Dynamics Simulations

**DOI:** 10.3390/ijms231911779

**Published:** 2022-10-04

**Authors:** Shunwei Chen, Huajing Zhang, Yi Li, Tingfeng Chen, Hao Liu, Xiujun Han

**Affiliations:** School of Materials Science and Engineering, Qilu University of Technology (Shandong Academy of Sciences), Jinan 250353, China

**Keywords:** photothermal conversion, C=N double bond, rotation, perpendicular configuration, conical intersection, excited state dynamics simulations, TD-DFT

## Abstract

Molecular photothermal conversion materials are recently attracting increasing attention for phototherapy applications. Herein we investigate the excitation and de-excitation processes of a photothermal molecule (C1TI) that is among the recently developed class of small-molecule-based photothermal imines with superb photothermal conversion efficiencies (PTCEs) up to 90% and a molecule (M2) that is constructed by replacing the amino group of C1TI with an H atom, via excited-state dynamics simulations based on the time-dependent density functional theory (TD-DFT). The simulations reveal fast (<150 fs of average time) nonradiative decays of the lowest excited singlet (S_1_) state to a conical intersection (CI) with the ground (S_0_) state in high yields (C1TI: 93.9% and M2: 87.1%). The fast decays, driven by C=N bond rotation to a perpendicular structural configuration, are found to be barrierless. The slight structural difference between C1TI and M2 leads to drastically different S_0_-S_1_ energy surfaces, especially M2 features a relatively much lower CI (0.8 eV in energy) and much more decay energy (1.0 eV) to approach the CI. This work provides insights into the de-excitation mechanisms and the performance tuning of C=N enabled photothermal materials.

## 1. Introduction

Photothermal conversion materials, converting photon energy into heat, have received considerable attention for their great promise in phototheranostics, especially for tumor ablation [1,2,3,4]. They allow selective tumor removal with minimized side effects by precisely irradiating with controllable dosage as compared to the intolerable adverse effects of traditional treatments including chemotherapy and radiotherapy, and thus are attracting substantial research effort [4]. Photothermal materials can be divided into inorganic and organic ones. Inorganic photothermal materials, which have drawn much more attention than organic ones for phototheranostics, including metal and transition metal sulfide/oxide nanoparticles, generally feature advantages of high photo-absorption, good photostability, and superior photothermal conversion efficiencies, but they have typical drawbacks such as poor-biocompatibility and long-term toxicity [2,4]. By contrast, organic photothermal materials feature incomparable advantages of biocompatibility, biodegradability, structural modification, and tunable photophysical and photochemical features, hence becoming increasingly popular [5]. In this emerging research field, several strategies for acquiring organic photothermal agents have been proposed, including the engineering of molecular stacking [6], electron/charge transfer [7,8], and resonance energy transfer [9]. Recently, a pioneering work by Tang et al. showcased the high potential of intramolecular motion-induced photothermy, triggering increasing research attention [10,11]. As a typical example, Li and co-authors recently reported several small photothermal agents that feature a light-driven rotatable C=N double bond with varying lengths of alkyl chains and achieve a superb photothermal conversion efficiency (PTCE) as high as 90% [12]. The field of molecular machines that are promising for various applications, such as catalysis, self-assembly, and molecule-based electronics, is quickly developing, especially in the recent decade [13]. As a typical member of the rotary motors featuring a double-bond, the C=N bond containing imines are a relatively new type of photo-driven molecular rotor [14,15,16]. The work by Li extends the application of the C=N photo-switches to a new area [12].

To date, small photothermal agents possessing a high PTCE are rare, and the mechanisms underlying the photophysics of the report molecules are pending in-detail understanding in order to advance and/or diversify their functions [12]. Thus, we have performed a combination of static calculations and dynamics simulations to gain insights into their excitation and de-excitation processes. The surface hopping excited-state dynamics simulations based on the time-dependent density functional theory (TD-DFT) reveal fast (average time of 143 and 114 fs for C1TI and M2, respectively) nonradiative decays from the first excited singlet (S_1_) state to the crossing region (conical intersection, CI) with the ground (S_0_) state. These fast decays occur for 93.9% and 87.1% of trajectories that were studied for C1TI and M2, respectively, fairly rationalizing the high PTCE of C1TI and its derivatives. The decay is enabled by the out-of-plane rotation of the C=N bond. The CI geometry features an elongated C=N distance and a perpendicular configuration between the donor and acceptor fragment. The slight structural modification to C1TI (replacing the amino group on the donor with an H atom) greatly alters the electronic structures, photo-absorption, and S_1_–S_0_ potential energy surface. Importantly, the modification leads to a relatively much lowered CI (energetic position) and much higher dissipation energy for decaying from the S_1_ state to the CI. The notable difference in the relative energetic positions of the critical points shall greatly affect the photothermal performances of these two molecules. Our results provide a molecular-level understanding of the C=N facilitated non-radiative excited-state decay and the property tuning of C=N incorporated photothermal materials.

## 2. Computational Details

All ground (S_0_) and excited (S_1_) state properties were studied based on DFT and TD-DFT, respectively, as implemented in the Gaussian 16 software [17], utilizing the ɷB97XD long-range separated function [18]. Non-adiabatic excited-state dynamics simulations were performed using the fewest-switches surface hopping approach based on TD-DFT by the Newton-X 2.6 package [19], interfacing with the Gaussian 16 software. The def-TZVP basis set was adopted for optimizing the structures and calculating the vertical excitations, whereas the 6-31G basis set was chosen for performing the dynamics simulations and plotting the potential energy surfaces [20]. The nature of the stationary points was ascertained by frequency analysis based on the same level of theory. The simulations started from the S_1_ state, and decoherence correction was taken into consideration during the simulation [19]. A total duration time of 400 fs was applied unless the S_1_–S_0_ gap becomes ≤0.2 eV. Following the literature, such a very small S_1_–S_0_ gap was taken as a sign of approaching a CI region, due to the typical drawbacks of TD-DFT in dealing with strong coupling regions, especially the CI [21,22,23,24]. The CI geometry was ascertained by the complete active space self-consistent field (CASSCF) method with the 6-31G(d) basis set [25,26]. A chiral isomer for each of the studied structures exists but is not studied for the identity of the left and right side of the acceptor, which should lead to great similarities between the two isomers in their total energies, oscillator strengths, and also structural features. The conclusions for the studied isomer shall also stand for the other case. The successive decay from the CI to the S_0_ state was not considered. The non-radiative decay to the S_0_ state will lead to different isomers, whereas the isomerization is beyond the topic of this work. The absorption cross-section spectra were simulated by the nuclear ensemble approach based on 80 points for each state from S_1_ to S_6_. The initial conditions (geometries and velocities) were chosen following the Wigner distribution within an absorption region of 2.2 ± 0.1 and 2.4 ± 0.2 eV, which respectively correspond to 33 and 31 trajectories. The spatial distributions of photoexcitation generated electron and hole at the Franck-Condon (FC) geometry were plotted by the Multiwfn 3.6 software [5,27]

## 3. Results and Discussion

**Figure 1:**
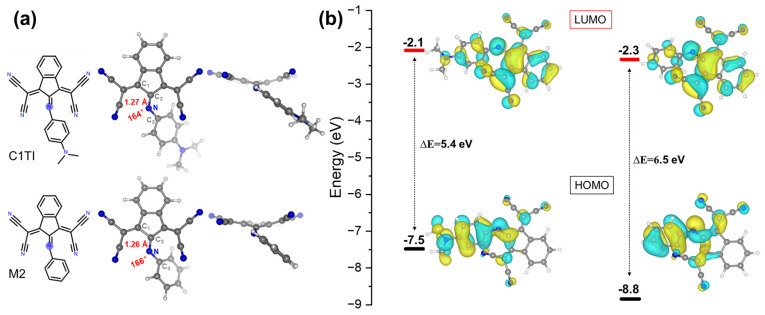
(**a**) Molecular structures and structural parameters of C1TI and M2 in the S_0_ state. (**b**) HOMO-LUMO energy levels and spatial distributions of the S_0_ state structure.

The S_0_ state structures of C1TI and M2 that we investigated are shown in Figure 1, having a slight difference at the donor part (a dimethylamino group or an H atom). To compare the change of structural features during the photo-excitation process, the lengths of the C=N bond connecting the donor and acceptor part and the C1-C2-N-C3 dihedral angles were measured. Structurally, C1TI and M2 show an overall similar appearance, that is, the donor and acceptor planes show a notable angle due to the imine group, and both structures show a cross-conjugated backbone that is important to their photophysical properties [28,29]. The dihedral angles are measured to be 164 and 166° for C1TI and M2, respectively, and the corresponding C=N bond lengths are 1.27 and 1.26 Å. For electronic structures, these two structures similarly feature the highest-occupied molecular orbital (HOMO) populated on the donor site while the lowest-unoccupied molecular orbital (LUMO) located on the acceptor, but the HOMO-LUMO energy gaps show a large difference of 1.1 eV. The S_0_–S_1_ vertical excitation energy of C1TI at the FC geometry is calculated to be 2.2 eV; by contrast, M2 shows a blue shift by 0.5 eV (Table S1). These apparent variations primarily ascribe to the decreased π-conjugation upon H replacement, implying the high tunability in the photo-absorption via π-network enlarging or reducing. Furthermore, the HOMO and LUMO, the main molecular orbital pair determining the S_0_–S_1_ transition (ππ*), located on different structural parts (donor and acceptor, respectively), in accordance with the intramolecular charge transfer character as reported (see also the electron-hole distributions upon photoexcitation in Figure S1) [12]. The HOMO-LUMO pair accounts for 86.8 and 83.7% of the S_0_–S_1_ transition respectively for C1TI and M2(the main orbital contributions for the S_2_–S_5_ excitations can be found in Table S1).

**Figure 2:**
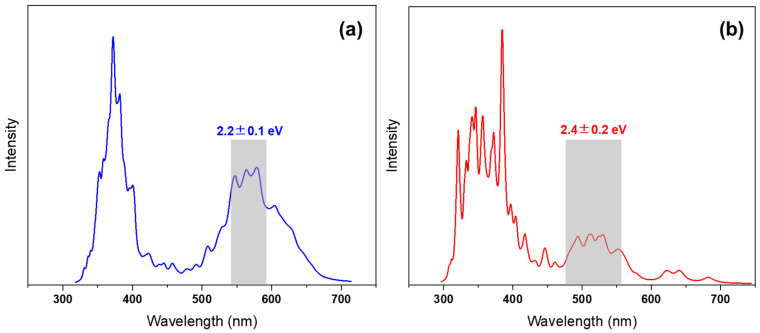
Absorption spectra of C1TI (**a**) and M2 (**b**), simulated based on the nuclear ensemble approach for the transitions of S_0_ → S_1_–S_6_. The shadows show the energy windows of the initial conditions selected for initiating the dynamics simulations.

Figure 2 shows the simulated absorption spectra of C1TI and M2 by considering the lowest six excitations (S_1_–S_6_) via the nuclear ensemble approach [30]. Though the simulated peaks blue shift as compared to the experimental results [12], the simulated spectra of C1TI capture the overall main experimental topographies, especially the relative intensity of the first and the second broad peak (located at ca. 375 and 560 nm, respectively). Such mismatch resulted due to the drawbacks of the theoretical method shall impart limited influence on our conclusions since we mainly focus on the qualitative mechanisms, other than quantitative values. In comparison, the structural modification leads to notable spectral variation, including the blue shifts in the excitations (see also the S_0_ → S_1–6_ vertical excitations in the FC region in Table S1). In practice, such a trivial structural alteration may impart vital influence on their photophysical performance, as also reflected in the blow results. To initiate the dynamics simulations, different starting geometries that correspond to the S_0_ → S_1_ transition were chosen within the energy window of 2.2 ± 0.1 and 2.4 ± 0.2 eV according to the Wigner distribution [31].

**Figure 3:**
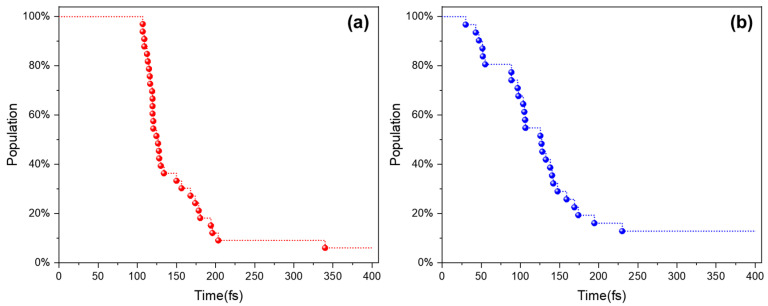
Percentage of the S_1_ states as a function of simulation time for C1TI (**a**) and M2 (**b**), respectively. Hopping to the S_0_ state was assumed to occur once the S_1_–S_0_ gap ≤ 0.2 eV. Within 400 fs total time for each simulation, 31 and 27 trajectories for C1TI and M2 successively reached the CI point, respectively. The blue and red dots represent trajectories stopped after reaching the CI at a given simulation time.

**Figure 4:**
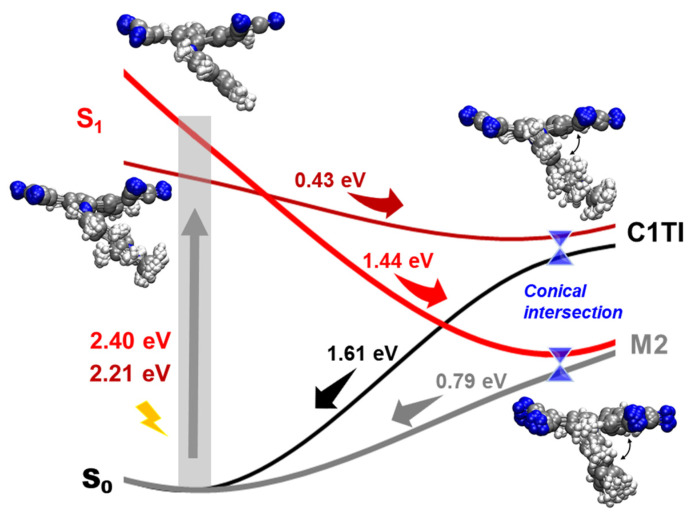
Schematics for the energetics of the nonradiative decay processes after photoexcitation from the S_0_ to S_1_ state for C1TI and M2, alongside the ensemble of all structures at the FC and CI point. The energies are averaged over all trajectories reaching the CI. The arrows on the structure ensemble indicate the rotation around the C=N bond towards a more perpendicular configuration.

The results of the dynamic simulations are summarized in Figure 3 (the simulation results of a representative trajectory are provided in Figures S2 and S3). Among the total 33 and 31 trajectories, 31 and 27 trajectories for C1TI and M2, respectively, finally stopped at the CI region, at which the S_1_–S_0_ energy gap is less than 0.2 eV (see Table S2 for details of each trajectory). For C1TI, its first trajectory takes 107 fs to reach the CI. The average time taken over all the CI-reached trajectories is calculated to be 143 fs. As a comparison, M2 shows a faster decay, though its initial geometries have higher S_1_–S_0_ gaps (2.2 ± 0.1 versus 2.4 ± 0.2 eV). For M2, the first trajectory reaching the CI takes 30 fs, with an average time taken of 114 fs. The faster decay of M2 is ascribed to the steeper S_1_ energy surface, as shown in Figure 4 for the relative energy positions of the S_0_ and S_1_ states at the FC and CI geometry, plotted by taking the average of each state over all the CI-involved trajectories. These results designate that the excited-state decay rate can be effectively tuned via structural modification. In practice, a slow decay offers more probability of intrinsic photophysical and/or photochemical processes competing with the nonradiative decay and consequently decreasing the PTCE. In Li’s work, the PTCE of C1TI nano-aggregates is measured to be 57.1% in the mixed solvents of tetrahydrofuran with phosphate-buffered saline at a concentration of 40 μM [12]. The fast decay of C1TI and M2 from the S_1_ state to the CI region in high yields (93.9% and 87.1%) suggests a low luminescent decay probability. In addition, a fast excited-state decay minimizes the loss of energy owning to intermolecular interactions. As an indication, the fast decay in C1TI and its analogs shall importantly contribute to their high PTCEs. It is clear that the nonradiative decay of excited states converts photon energy to heat, but whether the photoconversion involves the S_1_ → CI process and/or the CI → S_0_ process remains to be an open question. For the differences in the intramolecular motions and interactions with the surrounding micro-environments in the S_1_ and S_0_ states that influence the energy dissipation, the relative energy positions of CIs might be a critical factor governing the photothermal conversion performances of the materials.

**Figure 5:**
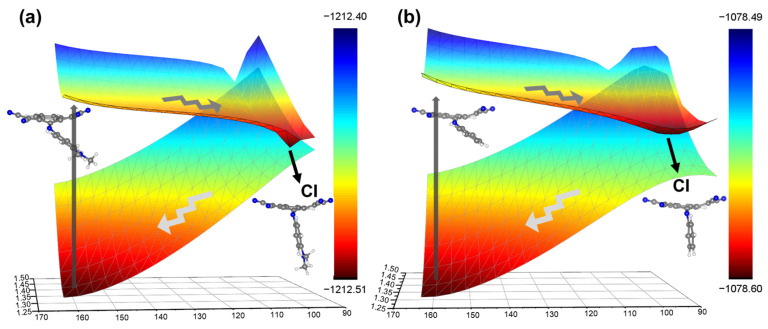
Potential energy surfaces of C1TI (**a**) and M2 (**b**) in the S_0_ and S_1_ states as a function of C1-C2-N-C3 dihedral angles and C2-N bond distance, obtained by performing a rigid scan based on the optimized S_0_ state structures(dihedral angles in ° and bond lengths in Å) at the ɷB97XD/6-31G level. The black and blue colors respectively represent the stable and unstable configurations with a given structural parameter(energy unit in a.u.).

**Figure 6:**
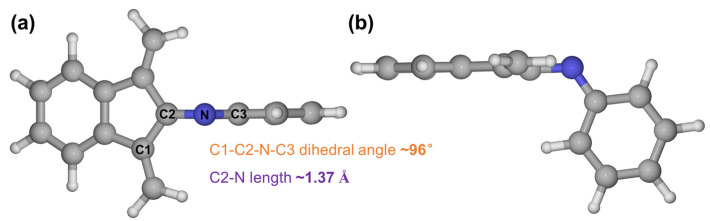
Top (**a**) and side (**b**) views of the CASSCF optimized CI structure of the simplified model. The dihedral angle for the C1-C2-N-C3 atoms is measured to be about 96°, while the C2-N bond length is about 1.37 Å.

Next, we discuss the main structural features of C1TI and M2 during the decay process. As shown in Figure 4 for the structure ensemble of all CI involved trajectories, the donor part overall rotates around the C=N bond along the clockwise direction, leading to decreased C1-C2-N-C3 dihedral angle (the average dihedral angles of C1TI and M2 at the CI are 113 and 109°, respectively). Actually, the rotation around the C=N bond to a perpendicular configuration, along with an elongated C-N bond, accounts for the CI formation [16,32]. This can be further understood from Figure 5, which shows the three-dimensional S_1_ and S_0_ potential energy surfaces that were plotted against the C=N bond length in the range of 1.25–1.50 Å and the C1-C2-N-C3 dihedral angles ranging from 90 to 170° by performing a rigid scan. Both the structures show the CI region in the dihedral angle range of 90–100°. The CI structure was further confirmed by the CASSCF calculations to a simplified model, showing basically the same structural feature as the TD-DFT obtained CI structure (Figure 6; Table S3).

As indicated by Figure 5, the S_1_ structures shall exclusively decay towards the CI after photoexcitation [33], as approaching CI from the S_1_ state at the FC geometry is barrierless, that is, no energy barrier exists for going across. We also attempted to optimize the S_1_ state minima imposing no structural constraint with TD-DFT but found no converged structure, where all attempts failed at structures with a small S_1_–S_0_ energy difference (<0.2 eV), in agreement with the barrierless feature of the decay process, as also reported in the literature [12]. Though our dynamics simulations have not investigated the process after S_1_–S_0_ surface crossing, that is, from the CI to S_0_, it can be inferred from Figure 5 that the unstable S_0_ state CI structure will downhill proceed towards the stable S_0_ structure. Such barrierless, exclusive decay processes are likely another reason contributing to the high PTCE of the reported molecules.

## 4. Conclusions

In summary, we have studied and compared the photophysical process of C1TI and M2 by performing both static calculations and dynamics simulations, primarily showing rationalization to the experimentally reported high PTCE performances of C1TI and its derivatives. Surface hopping non-adiabatic dynamics simulations based on TD-DFT reveal fast non-radiative decays of the S_1_ state to the CI region with the S_0_ state in a high yield (93.9% and 87.1% respectively for C1TI and M2). The fast decay process features a downhill potential energy surface towards the CI. Upon relaxation from the S_1_ state, structures of the two molecules rotate via the C=N bond to a more perpendicular configuration (between the donor and the acceptor fragment) when approaching the CI. The high-level theoretical method (CASSCF) further ascertains the perpendicular CI configuration. Notably, the small variation in the structures of C1TI and M2 leads to drastic different potential energy surfaces for their excitation and de-excitation. For specific, the CI of M2 is much lower (0.8 eV) in energy than that of C1TI, though M2 decreases from the S_1_ state in the FC region that is higher (0.2 eV) than that of C1TI. Such difference is likely to be critical to the photothermal conversion performances, especially given that it is elusive whether the S_1_ → CI and/or CI → S_0_ process is effective for photothermal conversion. This work provides a useful understanding of the de-excitation mechanisms and the property tuning of C=N facilitated photothermal materials.

## Data Availability

Not applicable.

## Supplementary Materials

The following supporting information can be downloaded at: https://www.mdpi.com/article/10.3390/ijms231911779/s1.
