# Supplementary material for: Revealing and Tuning the Photophysics of C=N Containing Photothermal Molecules: Excited State Dynamics Simulations
Source: Int J Mol Sci. 2022 Oct 4;23(19):11779. doi: 10.3390/ijms231911779 (PMC9570121; doi:10.3390/ijms231911779)
Supplement: Supplementary file 1 [file ijms-23-11779-s001.zip › ijms-1938214-supplementary.pdf]

Revealing and Tuning the Photophysics of C=N Containing Photothermal Molecules: Excited State Dynamics Simulations

Shunwei Chen*, Huajing Zhang, Yi Li, Tingfeng Chen, Hao Liu, Xiujun Han*
School of Materials Science and Engineering, Qilu University of Technology (Shandong Academy of Sciences), Jinan, China
*Corresponding author. Email: swchen@qlu.edu.cn (SW Chen), xjhan@qlu.edu.cn (XJ Han)

Supporting Information:

Table S1. Calculated energy (ΔE), oscillator strength (*f*), and the main molecular orbital (MO) contribution for the $S_0 \rightarrow S_1$-S_6 transitions at the ωB97XD/def-TZVP level of theory.

Transition	C1TI			M2		
	**ΔE** (eV)	***f*** (arb. unit)	**MO contribution**	**ΔE** (eV)	***f*** (arb. unit)	**MO contribution**
$S_0 \rightarrow S_1$	2.21	0.296	H→L 86.8%	2.72	0.080	H→L 83.7%
$S_0 \rightarrow S_2$	2.88	0.082	H-1→L+1 86.8%	3.54	0.048	H→L+1 82.6%
$S_0 \rightarrow S_3$	3.63	0.061	H-1→L 28.1%	3.74	0.061	H-1→L 76.0%
$S_0 \rightarrow S_4$	3.88	0.265	H-1→L 61.8%	3.95	0.055	H-2→L 70.6%
$S_0 \rightarrow S_5$	4.01	0.437	H-2→L+1 76.0%	4.03	0.446	H-1→L+1 49.1%
$S_0 \rightarrow S_6$	4.07	0.012	H-3→L 71.8%	4.30	0.053	H-4→L 31.5%

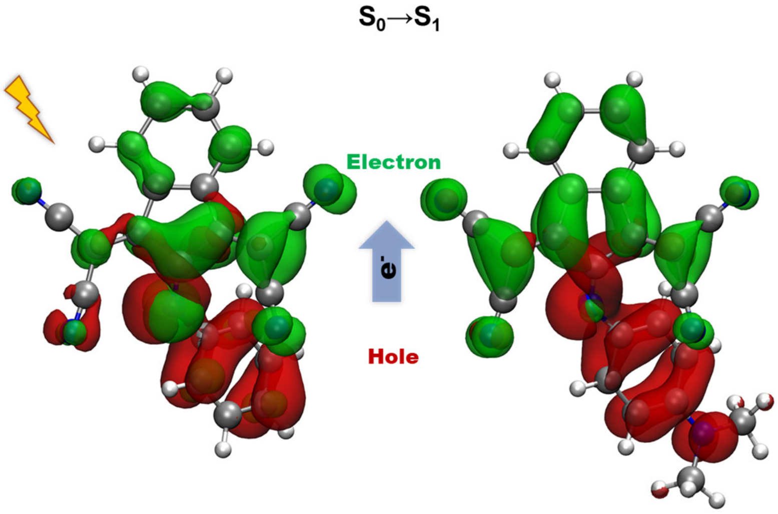

Figure S1 Photoexcited electron and hole at the Franck-Condon (FC) geometry for C1TI and M2. The red nodes represent the populating site of the hole, whereas the green ones denote the electron.

Table S2. Time-taken for each of the CI-reached trajectories.

C1TI			M2		
**No.**	**Time-taken**	**S_1-S_0 gap**	**No.**	**Time-taken**	**S_1-S_0 gap**
1	107	0.194	1	30	0.144
2	107	0.167	2	43	0.191
3	109	0.162	3	47	0.189
4	109	0.130	4	51.5	0.172
5	112.5	0.145	5	52	0.192
6	113.5	0.150	6	55	0.180
7	115	0.159	7	88.5	0.155
8	116	0.180	8	88.5	0.177
9	116.5	0.184	9	96.5	0.117
10	119	0.180	10	97.5	0.164
11	119.5	0.192	11	103.5	0.169
12	119.5	0.177	12	105	0.157
13	119.5	0.173	13	106	0.166
14	120.5	0.183	14	106.5	0.143
15	120.5	0.191	15	125.5	0.169
16	124.5	0.188	16	127	0.171
17	126.5	0.179	17	128	0.183
18	127.5	0.178	18	132.5	0.130
19	128	0.130	19	138	0.181
20	130	0.140	20	140.5	0.129
21	134	0.200	21	142	0.157
22	150	0.151	22	147.5	0.190
23	156.5	0.176	23	159	0.155
24	168	0.197	24	169	0.162
25	174	0.198	25	174	0.123
26	178.5	0.172	26	194.5	0.172
27	180.5	0.158	27	230	0.188
28	194	0.198	28	400	1.654
29	196	0.186	29	400	3.022
30	203.5	0.179	30	400	2.448
31	340	0.191	31	400	2.275
32	400	2.213			
33	400	1.511			

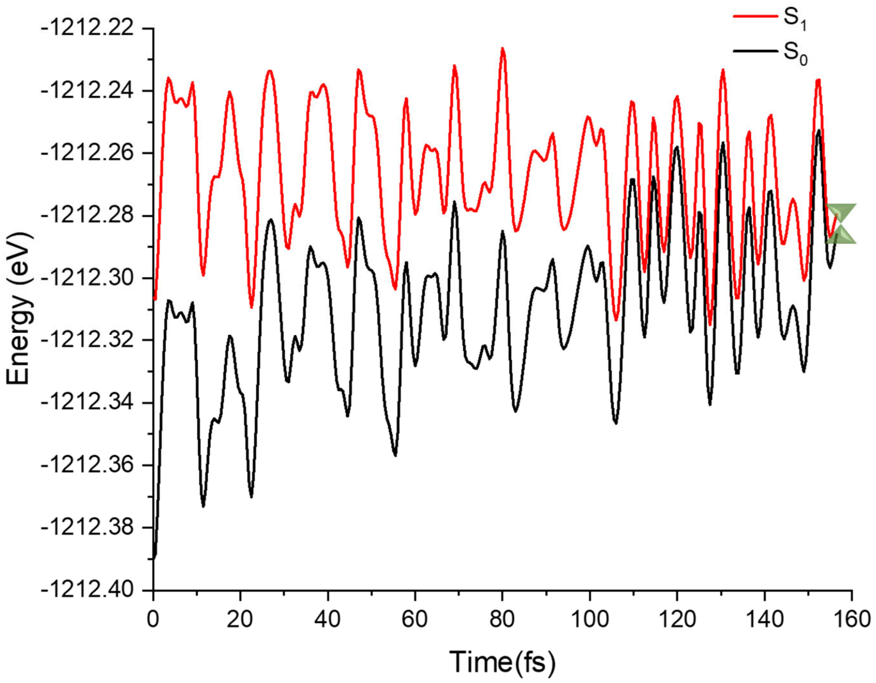

Figure S2 Energy evolution of the S_1 and S_0 state during the simulation for a representative trajectory. This trajectory reached a CI at the time of 156.5 fs.

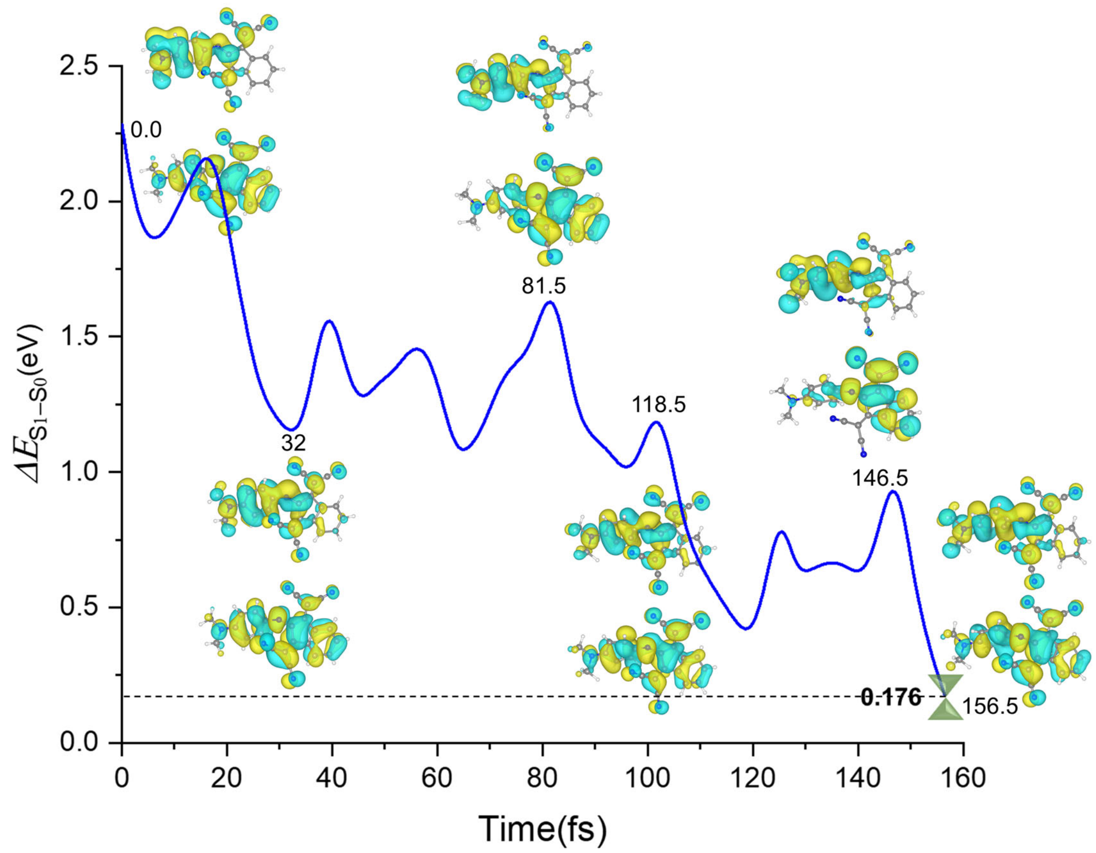

Figure S3 Evolution of the S_1-S_0 energy gap ($\Delta E_{S1\text{-}S0}$) during the simulation for the representative trajectory (Fig. S2). The simulation stopped after $\Delta E_{S1\text{-}S0} \leq 0.20$ eV (0.176 eV). The HOMO (upper) and the LUMO (lower) at several time points are also shown.

Table S3. Cartesian coordinates of the CASSCF optimized CI structure for the simplified model.

Atoms	X	Y	Z
C	-0.0694160	-0.0367980	0.0207780
C	-0.0821030	-0.0335560	1.4127680
C	1.0982670	-0.1080260	2.1277100
C	2.2950440	-0.1903990	1.4338260
C	2.3073340	-0.1940920	0.0446140
C	1.1231900	-0.1161950	-0.6713530
C	-1.4654490	0.0682930	-0.4920470
C	-2.3119560	0.0222560	0.6613640
C	-1.4882170	0.0828480	1.8853220
H	1.0948130	-0.1016170	3.2024650
H	3.2221410	-0.2481310	1.9744000
H	3.2440100	-0.2548410	-0.4790310
H	1.1394360	-0.1159110	-1.7460070
C	-1.8567740	0.1568840	-1.7920400
N	-3.6725500	0.1330430	0.6486930
C	-4.4996970	-0.9206110	0.6478600
C	-4.0512560	-2.2755120	0.6149740
C	-5.9081640	-0.6802300	0.6725040
C	-4.9666810	-3.3192090	0.6116070
H	-2.9927600	-2.4820780	0.5913330
C	-6.8073810	-1.7315780	0.6701070
H	-6.2426840	0.3456170	0.6917220
C	-6.3471140	-3.0625490	0.6400650
H	-4.6073110	-4.3352110	0.5867420
H	-7.8655380	-1.5290960	0.6901120
C	-1.9231280	0.2254340	3.1237120
H	-1.1404670	0.1703980	-2.5882950
H	-2.8892120	0.2394010	-2.0575750
H	-2.9672200	0.3210610	3.3459800
H	-1.2412010	0.2652310	3.9525350
H	-7.0499580	-3.8800370	0.6370930
